# Particulate Hexavalent Chromium Inhibits RAD51 Paralogs Necessary for RAD51 Filament Formation and Stabilization During Homologous Recombination Repair

**DOI:** 10.3390/occuphealth1010013

**Published:** 2026-03-23

**Authors:** Aggie R. Williams, Idoia Meaza, Haiyan Lu, James T. F. Wise, Sandra S. Diven, Jennifer H. Toyoda, J. Calvin Kouokam, John Pierce Wise

**Affiliations:** 1 Wise Laboratory of Environmental and Genetic Toxicology, Department of Pharmacology and Toxicology, University of Louisville, Louisville, KY 40202, USA; 2 Wise Laboratory of Nutritional Toxicology & Metabolism, Department of Human Nutrition, Food and Animal Sciences, College of Tropical Agriculture and Human Resilience, University of Hawai’i, Honolulu, HI 96822, USA

**Keywords:** lung cancer, metal exposure, hexavalent chromium, mechanisms of carcinogenesis, DNA repair inhibition, RAD51 paralogs

## Abstract

Hexavalent chromium [Cr(VI)] is a lung carcinogen. Central to its carcinogenic mechanism are Cr(VI)-induced DNA double strand breaks and chromosome instability. While breaks are usually repaired in healthy cells, Cr(VI) inhibits homologous recombination repair by targeting RAD51. RAD51 paralogs (RAD51B, RAD51C, RAD51D, XRCC2, and XRCC3) are responsible for RAD51 loading and the stabilization of nucleoprotein filaments necessary for DNA strand exchange and repair. This study aimed to investigate the effects of Cr(VI) exposure on RAD51 paralogs. WTHBF-6 cells, a human lung cell line, were exposed to various environmentally and occupationally relevant concentrations of zinc chromate for acute (24 h) and prolonged (120 h) exposure times. After exposure to Cr(VI), we collected RNA for sequencing and assessed the ability of DNA repair proteins to form foci using immunofluorescence. Protein levels were measured with western blotting, RNA-Seq was validated with RT-qPCR, and protein–protein interactions were assessed with the Proximity Ligation Assay (PLA) assay. Cr(VI) transcriptionally repressed all RAD51 paralogs. Further functional analyses showed that Cr(VI) inhibited the foci formation of RAD51D after acute and prolonged exposures and of XRCC2 and XRCC3 after prolonged exposure. Cr(VI) also inhibited overall RAD51D protein expression, as well as its interaction with RAD51. These findings suggest that Cr(VI) inhibits all RAD51 paralogs, but RAD51D might be an early target of Cr(VI), leading to the loss of RAD51 filament formation and function and the overall inhibition of homologous recombination repair.

## 1. Introduction

Occupational and environmental lung carcinogens are a human health threat. For example, exposure to asbestos causes malignant pleural mesothelioma, a rare but aggressive form of cancer in the lungs that is challenging to treat as it relies on surgery, systemic therapy, and radiotherapy [1]. One of the factors driving the lack of more specialized treatments for these cancers relies on the lack of our understanding of the mechanisms of toxicant-induced lung carcinogenesis [2–6]. For example, a recent study suggests the potential contribution of a metal lung carcinogen, hexavalent chromium [Cr(VI)], to the carcinogenicity of chrysotile asbestos [7]. These recent findings highlight the need to further explore how metals cause lung cancer to develop targeted therapeutic and preventative strategies.

Among lung carcinogens, metals are a key group with significant occupational and environmental exposure risks and represent a major cause of human lung cancer [8,9]. Indeed, five metals, i.e., nickel, cadmium, arsenic, beryllium, and hexavalent chromium [Cr(VI)], are on the list of the eight most dangerous lung carcinogens in occupational settings [10]. Of these, Cr(VI) is of special concern, as among the five metals, Cr(VI) had the highest attributable risk to cause lung cancer due to its high prevalence in occupational settings [11]. Further, the European Commission’s occupational cancer risk assessment ranked Cr(VI) among the top three agents contributing to cancer deaths in Europe [12]. In addition, a study showed that the attributable risk for Cr(VI)-induced lung cancer in welders was 64%, which means that from 110 million workers exposed to welding fumes worldwide, 70.4 million are likely to develop lung cancer [13,14]. Cr(VI) is clearly a potent occupational lung carcinogen; however, Cr(VI) is found in particulate matter, tobacco smoke, e-cigarettes, burning fossil fuels, and airborne microplastics, among other sources, and thus its contributions to lung cancer through environmental exposures should not be overlooked [15–21].

Despite Cr(VI) contributing to the lung cancer burden, the mechanisms by which it causes this disease are unclear. Cr(VI)-induced chromosome instability is a key driving factor of how Cr(VI) induces tumors [22–25]. DNA double strand breaks are the underlying lesions that progress to structural chromosome instability if they are left unrepaired or are repaired through error-prone DNA repair pathways. Many laboratories, including our own, have shown that Cr(VI) induces DNA double strand breaks in human lung cells [26–33]. Importantly, these key lesions occur during the late S and G2 phases of the cell cycle, where homologous recombination repair is most active [29,30]. Previous work from our laboratory has also shown that acute (24 h) exposure to Cr(VI) induces canonical homologous recombination repair, while prolonged exposures (>48 h) inhibit this pathway, leading to an increase in structural chromosome instability [26,28,32,34,35].

The inhibition of homologous recombination repair mediated by Cr(VI) has been characterized by a decrease in RAD51 filament formation, its ability to repair foci, decreased nuclear localization, and a decrease in homology-directed repair ability using a reporter assay [26,28,32,35,36]. To function in homologous recombination, RAD51 first loads onto a single-stranded DNA to form a helical nucleoprotein filament structure that is crucial for carrying out the homology search and sister chromatid strand invasion to perform the repair [37–40]. Notably, Cr(VI) reduces both the number of RAD51 nucleoprotein filaments and their structural complexity, and this loss of RAD51 filaments results in impaired homologous recombination repair [32]. However, how Cr(VI) impacts the proteins involved in the loading and stabilization of RAD51 on these filaments is unknown.

The loading of RAD51 onto the filament is mediated by a family of proteins called RAD51 paralogs. RAD51 paralogs are proteins that share structural similarities with RAD51 [41,42]. There are five RAD51 paralogs, including RAD51B, RAD51C, RAD51D, X-ray repair cross-complementing 2 (XRCC2), and XRCC3 [43,44]. These paralogs assist in different steps of RAD51 regulation, including the formation, stabilization, and elongation of RAD51 filaments. They function within multi-protein complexes: (1) the BCDX2 complex (comprising RAD51B, RAD51C, RAD51D, and XRCC2) and (2) the CX3 complex (comprising RAD51C and XRCC3) [43,45]. These two complexes act during different stages of the homologous recombination repair pathway. The BCDX2 complex functions before RAD51 recruitment and plays a crucial role in the formation and stabilization of RAD51 filaments [46–49]. In contrast, the CX3 complex acts downstream of RAD51 recruitment, facilitating remodeling, stability, and strand invasion of the RAD51 nucleoprotein filament [47,50–52]. While the effects of Cr(VI) on the paralogs are unknown, previous studies show increased cytotoxicity and chromosome instability in XRCC3- or RAD51C-deficient Chinese Hamster Ovary (CHO) cells [53,54]. These two studies indicate that RAD51 paralogs are important in the response to Cr(VI). However, whether Cr(VI) targets RAD51 paralogs to inhibit the loading and stabilization of RAD51 filaments is unknown. This study addresses this important data gap by investigating the impact of particulate Cr(VI) exposure on RAD51 paralogs in the BCDX2 and CX3 complexes.

## 2. Materials and Methods

### 2.1. Chemicals and Reagents

The information about the chemicals and reagents used in this study is provided in the Supplementary Materials.

### 2.2. Cell Culture

WTHBF-6 cells are an hTERT-immortalized clonal cell line derived from human bronchial fibroblasts. This cell line exhibits normal growth parameters and a normal, stable karyotype and is non-tumorigenic in immunocompromised mice [55]. Cells were maintained in Dulbecco’s Modified Eagle Medium/Nutrient Mixture F-12 (DMEM/F-12) supplemented with 15% Cosmic calf serum (CCS), 0.2 mM Glutagro, 0.1 mM sodium pyruvate, and 1% penicillin/streptomycin and fed every 48 h. WTHBF-6 cells were sub-cultured every three to four days. WTHBF-6 cells were maintained in a 37 °C, humidified incubator with 5% CO_2_. Our cells are routinely screened for authenticity, including karyotyping when cells are thawed and after 3 months of continuous culture and short tandem repeat analysis performed at approximately one-year intervals by the American Type Culture Collection (Manassas, VA, USA).

### 2.3. Zinc Chromate Preparation

As previously described, zinc chromate was prepared as a suspension of particles in cold double-distilled water in a borosilicate scintillation vial, stirred overnight with a magnetic stir bar at 4 °C [35]. We found that this method produces zinc chromate particles with a diameter range of 1.7–2.6 μm and a mean diameter of 2.3 μm [35]. During the administration and preparation of the dilutions, particles were kept in suspension using a vortex mixer. The dilutions were dispensed directly into cultures from these suspensions. Final zinc chromate concentrations in the cell culture ranged from 0 to 0.3 μg/cm^2^. This range reflects levels that are relevant to human exposures and is lower than typical occupational exposure limits and representative of potential environmental contamination [56,57].

### 2.4. RNA Sequencing

WTHBF-6 cells were exposed to 0.1, 0.2, and 0.3 μg/cm^2^ zinc chromate for either 24 h or 120 h. After treatment, cells were collected and rinsed with cold PBS. Total RNA was isolated using the MagMAX mirVana Total RNA Isolation Kit (Thermo Fisher Scientific, Waltham, MA, USA), following the manufacturer’s protocol, using acid phenol:chloroform extraction. Three independent experiments (three independent biological replicates) were performed. This method has been described in our previous publications [57,58]. RNA sequencing was carried out by the University of Louisville Genomics Facility as previously described [57]. Sequencing was performed on an Illumina NextSeq 500 (Illumina Inc., San Diego, CA, USA) using NextSeq 500/500 75 cycles High Output Kit v2.5 (20024906). Differential expression analysis was performed using the DESeq2 tool as previously described [57]. Heatmaps were generated using Partek^™^ Flow^™^ software (Version 11.0). Gene names were translated into ENSEMBL gene IDs, and data were uploaded to the Partek software. The heatmaps display the log2 (fold change) count data organized in the heatmap through hierarchical clustering. The scale was set to 1 and −1.

### 2.5. Reverse Transcriptase Quantitative Polymerase Chain Reaction (RT-qPCR)

The validation of RNA-Seq results was performed using RT-qPCR as previously described with minor alterations [57]. Cells were seeded on 100 mm dishes and treated with zinc chromate. Briefly, cells were lysed directly in culture plates and homogenized. Total RNA was extracted as described above. The RNA quality and concentration were measured using a Nanodrop ND-1000 spectrophotometer (Nanodrop Technologies, Wilmington, DE, USA). cDNA synthesis was performed using a High-Capacity cDNA Reverse Transcription Kit according to the manufacturer’s instructions with slight modifications. Specifically, a 2X master mix was prepared using random primers and combined with 2 μg total RNA (per 20 μL reaction). TaqMan RNA primers (Hs00864094_m1; RAD51D-Hs00979562_m1; GAPDH-Hs027899) were mixed with TaqMan Universal PCR Master mix II and cDNA in triplicate. Controls without RNA, reverse transcriptase, and/or cDNA were included in all RT-qPCR runs. RT-qPCR used a StepOne Plus Real-Time Polymerase Chain Reaction (RT-qPCR) machine. The ΔΔCt method was employed, with untreated (0 μg/cm^2^ zinc chromate) cells as the control sample for each timepoint and GAPDH as an endogenous control.

### 2.6. Immunofluorescence

Immunofluorescence was performed to identify protein foci as previously described, with minor alterations [59]. WTHBF-6 cells were seeded on 4-well glass chamber slides precoated with AthenaES^®^ FNC (Athena enzyme systems, Baltimore, MD, USA) coating mix and allowed to re-enter logarithmic growth for 48 h before treatment with zinc chromate for 24 or 120 h. After zinc chromate treatments, cells were either fixed with 4% paraformaldehyde for 5 or 10 min, permeabilized with 0.2% or 0.5% Triton X-100 for 5 or 30 min, and blocked with either 10% goat serum and 5% bovine serum albumin (BSA) or 4% BSA in phosphate-buffered saline (PBS) for 30 min or 1 h. Cells were then incubated with primary antibodies targeting RPA (1:100), RAD51B (1:50), RAD51C (1:250), RAD51D (1:500), XRCC2 (1:100), and XRCC3 (1:500) overnight at 4 °C. Cells were washed with PBS and incubated with Alexa Fluor 488 or Alexa Fluor 594 for 1 h in the dark. Cells were washed with PBS, and coverslips were mounted with ProLong Diamond antifade mountant with DAPI. Nuclear foci were scored visually in 100 cells per condition per timepoint using fluorescent microscopy. Results were expressed as the total number of foci, so that untreated controls had less than 5% of cells with this level of foci, or as the total number of foci in 100 cells.

### 2.7. Western Blot Analysis

Western blot was performed as previously described with minor alterations [60]. Briefly, cells were seeded on 100 mm dishes and treated with zinc chromate. At the end of the treatment period, i.e., after 24 h or 120 h, media were removed, and cells were rinsed with PBS and released from the plate with 0.25% trypsin-EDTA. Cell pellets were collected in 1.5 mL microfuge tubes. Whole-cell extracts were obtained by washing, collecting, and incubating cells in 500 μL of extraction buffer Pierce RIPA Buffer with protease and phosphatase inhibitors at 100× for 20 min on ice. Cells were centrifuged at 14,000 rpm for 10 min. The supernatant was collected and stored at −80 °C. Immunoblots were probed with anti-RAD51D (1:1000). Equal loading was confirmed based on alpha-tubulin (1:1000) detection. Samples were run using 4–15% Mini-PROTEAN^®^ TGX^™^ (BioRad, Berkley, California, USA) precast protein gels and transferred to 0.45 μm nitrocellulose membranes. Immunoblots were incubated with IRDye 800 and 680 secondary antibodies, and fluorescence was detected and quantified using an Odyssey Imager (LI-COR Biosciences, Lincoln, NE, USA).

### 2.8. Duolink^™^ Proximity Ligation Assay (PLA)

The Duolink^™^ Proximity Ligation Assay (PLA) (Merck KGaA, Darmstadt, Germany) was performed following the manufacturer’s protocol. Briefly, cells were seeded in 8-well chamber slides and allowed 48 h to resume logarithmic growth. Cells were then treated with zinc chromate for either 24 or 120 h. After treatment, the cells were fixed, permeabilized, and blocked with a blocking buffer.

To prepare for the PLA reaction, as the DuolinkTM PLA fluorescence protocol instructed, slides were covered with DuolinkTM blocking solution and incubated in a heated humidity chamber for 60 min at 37 °C. Primary antibodies were added and incubated again at 4 °C. Next, slides were incubated with PLUS and MINUS PLA probes (1:5) for 1 h at 37 °C and ligation buffer and ligase (diluted 1:5 and 1:40) for 30 min at 37 °C. Amplification buffer and polymerase (diluted 1:5 and 1:80) were added for 100 min at 37 °C. Slides were washed with 1X DuolinkTM wash buffer A between incubations. After the last incubation, slides were washed with 1X DuolinkTM wash buffer B, dried, cover-slipped with DuolinkTM DAPI, and stored at 4 °C. Slides were then imaged using a 20× objective on a Keyence BZ-X8000, with quadrants of 6 by 6 images obtained and stitched together, where at least 100 cells per concentration/timepoint were scored and analyzed using the BZ-X800 Viewer software (v. 1.3.0) and BZ-X800 Analyzer software (V.1.1.2) (Keyence Corporation, Elmwood Park, NJ, USA).

### 2.9. Statistics

Results are expressed as the mean ± standard error of the mean (SEM) from at least 3 independent experiments (biological replicates). Statistics were performed as previously described [61]. Data was tested for normality, and for parametric data, a *t*-test with Welch’s correction was used, while for non-parametric data, the Mann–Whitney test was used. Statistical significance was determined at a *p*-value lower than 0.05. However, we also included an additional level of *p* < 0.1 to show effects that have a 90% chance of being significant. Tests were performed in the GraphPad Prism 10 software (GraphPad Software, La Jolla, CA, USA).

## 3. Results

### 3.1. Particulate Cr(VI) Exposure Inhibits the Transcription of Genes Involved in the BCDX2 and CX3 Complexes

**Figure 1.**
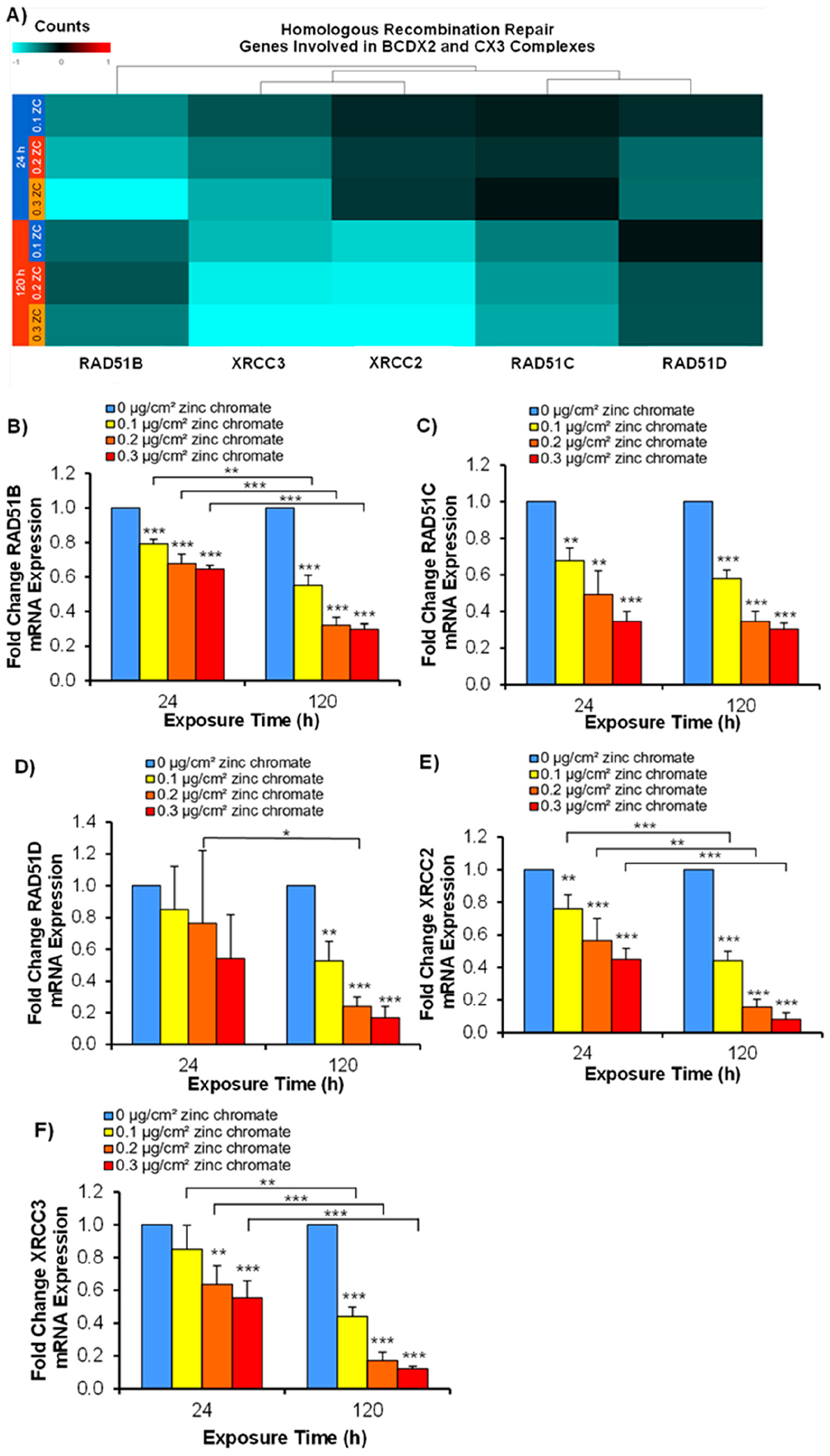
Particulate Cr(VI) transcriptionally represses genes in the BCDX2 and CX3 complexes. Data represent the average fold change in mRNA expression from three independent experiments ± standard error of the mean. * Statistically significant compared to control (*p* < 0.1); ** statistically significant compared to control (*p* < 0.05); *** statistically significant compared to control (*p* < 0.01); brackets show timepoint comparisons. (**A**) The heatmap shows the log2(fold change) data for RAD51B, RAD51C, RAD51D, XRCC2, and XRCC3. The scale has been set to 1 and −1 for ease of comparison across conditions and genes. (**B**–**F**) Validation of RNA-seq using RT-qPCR. (**B**) RAD51B, (**C**) RAD51D, (**D**) RAD51C, (**E**) XRCC2, and (**F**) XRCC3.

We first investigated how Cr(VI) exposure affects the expression patterns of the BCDX2 and CX3 complexes via RNA-Seq. Results showed that Cr(VI) exposure downregulates all genes involved in these complexes to some extent. Interestingly, there were distinct patterns of differential expression across the RAD51B, RAD51C, RAD51D, XRCC2, and XRCC3 genes, as shown in a hierarchical clustering heatmap (Figure 1A). After 24 h of exposure to zinc chromate, RAD51B, XRCC3, and RAD51D were most repressed followed by XRCC2 and RAD51C. However, after 120 h of exposure to zinc chromate, there was a decrease in expression found in XRCC3, XRCC2, and RAD51C followed by RAD51B and RAD51D.

We then confirmed the trends in the expression of these paralogs using reverse transcription-quantitative polymerase chain reaction (RT-qPCR) following particulate Cr(VI) exposure. The results showed that particulate Cr(VI) reduced RAD51B, RAD51C, RAD51D, XRCC2, and XRCC3 mRNA levels in a concentration-dependent manner after both 24 and 120 h of exposure (Figure 1B–F). RAD51B mRNA levels were reduced to 0.79, 0.68, and 0.65 relative to the control after 24 h of exposure to 0.1, 0.2, and 0.3 μg/cm^2^ zinc chromate, respectively. RAD51C mRNA levels were reduced to 0.68, 0.49, and 0.35 relative to the control after 24 h of exposure to 0.1, 0.2, and 0.3 μg/cm^2^ zinc chromate, respectively. RAD51D mRNA levels were reduced to 0.85, 0.76, and 0.54 relative to the control after 24 h of exposure to 0.1, 0.2, and 0.3 μg/cm^2^ zinc chromate, respectively. XRCC2 mRNA levels were reduced to 0.76, 0.56, and 0.45 relative to the control after 24 h of exposure to 0.1, 0.2, and 0.3 μg/cm^2^ zinc chromate, respectively. XRCC3 mRNA levels were reduced 0.85, 0.64, and 0.56 relative to the control after 24 h of exposure to 0.1, 0.2, and 0.3 μg/cm^2^ zinc chromate, respectively. These reductions persisted after 120 h, with mRNA levels decreasing further, where, after this prolonged exposure, RAD51B, RAD51C, RAD51D, and XRCC2 mRNA levels were significantly reduced at all concentrations compared to the control group. Specifically, RAD51B mRNA levels were reduced to 0.55, 0.32, and 0.3 relative to the control after 120 h of exposure to 0.1, 0.2, and 0.3 μg/cm^2^ zinc chromate, respectively. RAD51C mRNA levels were reduced to 0.58, 0.35, and 0.30 relative to the control after 120 h exposure to 0.1, 0.2, and 0.3 μg/cm^2^ zinc chromate, respectively. RAD51D mRNA levels were reduced to 0.53, 0.24, and 0.17 relative to the control after 120 h of exposure to 0.1, 0.2, and 0.3 μg/cm^2^ zinc chromate, respectively. XRCC2 mRNA levels were reduced to 0.44, 0.15, and 0.08 relative to the control after 120 h of exposure to 0.1, 0.2, and 0.3 μg/cm^2^ zinc chromate, respectively. XRCC3 mRNA levels were reduced to 0.45, 0.17, and 0.12 relative to the control after 120 h of exposure to 0.1, 0.2, and 0.3 μg/cm^2^ zinc chromate, respectively.

### 3.2. Particulate Cr(VI) Inhibits Foci of Paralogs Involved in the CX3 Complex

**Figure 2.**
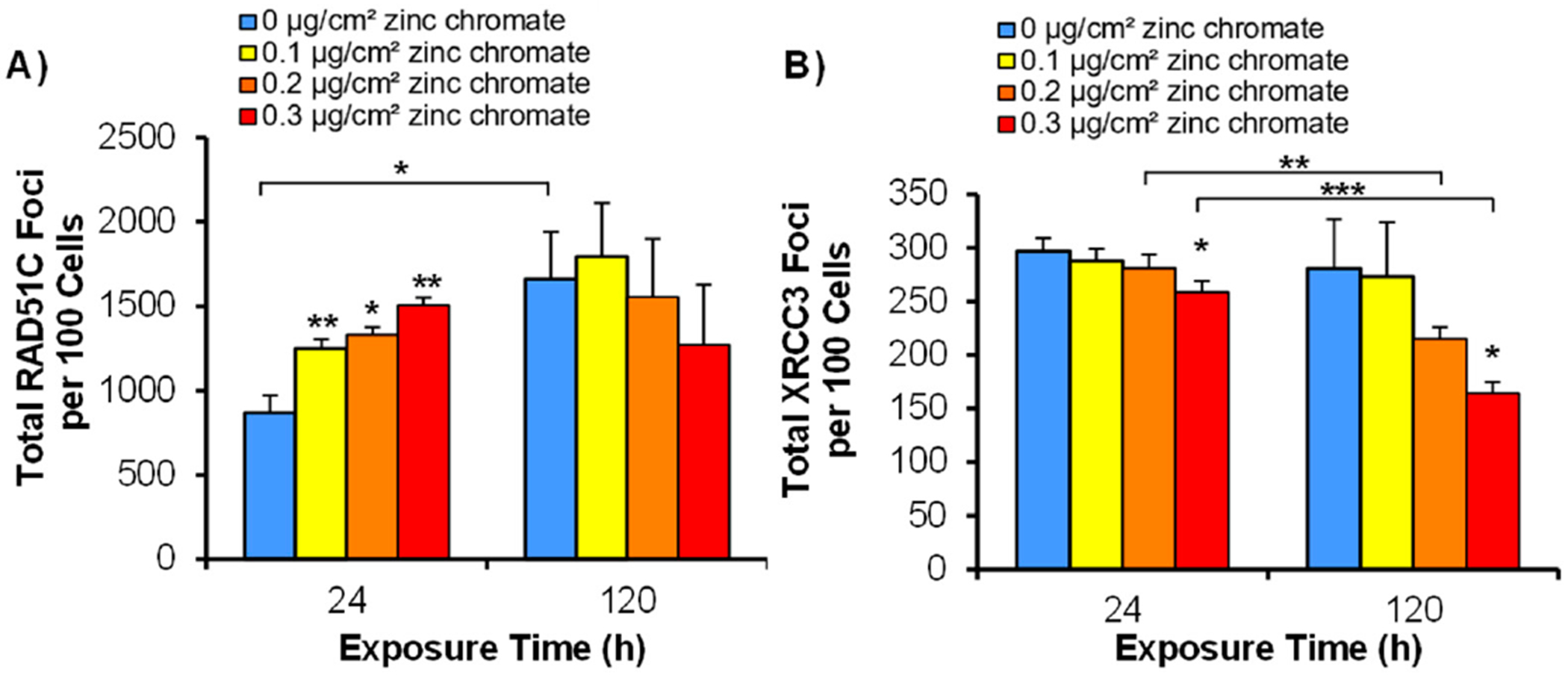
Particulate Cr(VI) exposure inhibits XRCC3, a key component of the CX3 complex. The figure shows that acute exposure to zinc chromate induces RAD51C, while both acute and prolonged zinc chromate exposures inhibit XRCC3 foci. Data represent the average from three independent experiments ± standard error of the mean. * Statistically significant compared to control (*p* < 0.1); ** statistically significant compared to control (*p* < 0.05); *** statistically significant compared to control (*p* < 0.01); brackets show timepoint comparisons. (**A**) Total RAD51C foci in 100 cells. (**B**) Total XRCC3 foci in 100 cells.

The CX3 complex acts downstream of RAD51 recruitment, facilitating remodeling, stability, and strand invasion of the RAD51 nucleoprotein filament. Given our previous findings that Cr(VI) reduced the amount and complexity of RAD51 nucleoprotein filament formation [32], we hypothesized that disrupting the CX3 complex may result in fewer filaments due to a lack of stabilization. For RAD51C foci formation, we found that 24 h of particulate Cr(VI) exposure increased RAD51C foci formation. Specifically, 0.1, 0.2, and 0.3 μg/cm^2^ zinc chromate induced 869, 1250, 1329, and 1508 total RAD51C foci, respectively (Figure 2A). After 120 h of exposure to 0, 0.1, 0.2, and 0.3 μg/cm^2^ zinc chromate, it produced 1661, 1795, 1555, and 1272 total foci, respectively (Figure 2A).

We found that 24 h of particulate Cr(VI) exposure did not increase XRCC3 foci formation and yielded levels similar to controls, though there was a small but significant reduction at 0.3 μg/cm^2^ zinc chromate. Specifically, 0.1, 0.2, and 0.3 μg/cm^2^ zinc chromate produced 297, 288, 281, and 258 total foci, respectively (Figure 2B). After 120 h of exposure, XRCC3 foci formation was significantly inhibited at both 0.2 and 0.3 μg/cm^2^ zinc chromate (Figure 2B). Specifically, 0.1, 0.2, and 0.3 μg/cm^2^ zinc chromate produced 281, 273, 215, and 164 total foci, respectively (Figure 2B).

### 3.3. Particulate Cr(VI) Inhibits Foci of Paralogs That Form the BCDX2 Complex

**Figure 3.**
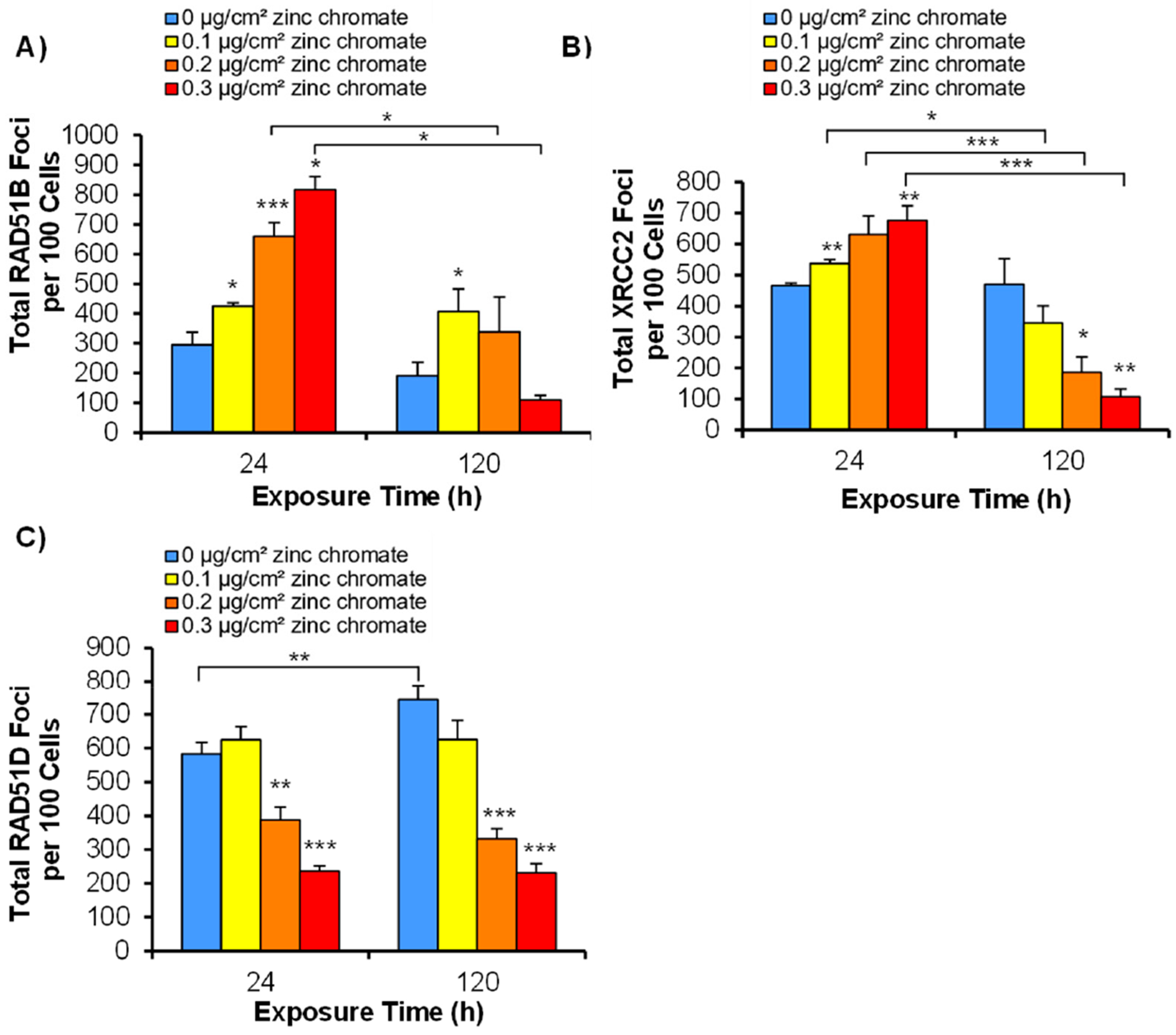
Particulate Cr(VI) exposure inhibits RAD51D and XRCC2, both key components of the BCDX2 complex. The figure shows that acute exposure to zinc chromate increases RAD51B and XRCC2 foci, while prolonged exposures inhibit them, with both acute and prolonged exposures inhibiting RAD51D. Data represent the average from three independent experiments ± standard error of the mean. * Statistically significant compared to control (*p* < 0.1); ** statistically significant compared to control (*p* < 0.05); *** statistically significant compared to control (*p* < 0.01); brackets show timepoint comparisons. (**A**) Total RAD51B foci in 100 cells. (**B**) Total RAD51D foci in 100 cells. (**C**) Total XRCC2 foci in 100 cells.

As mentioned above, we found that RAD51C foci formation increased following acute Cr(VI) exposure but was inhibited after prolonged exposure (Figure 3). Here, we considered the effect of Cr(VI) on the remaining members of the BCDX2 complex, i.e., RAD51B, RAD51D, and XRCC2, as they are essential in the loading of RAD51 onto the filament. We found that RAD51B foci formation increased after 24 h of zinc chromate exposure as 0, 0.1, 0.2, and 0.3 μg/cm^2^ zinc chromate produced 295, 425, 660, and 817 total foci, respectively (Figure 3A). After 120 h of Cr(VI) exposure, total foci were greater than the control at the two lower treatments (0.1 and 0.2 μg/cm^2^ zinc chromate) but were reduced at the highest treatment of 0.3 μg/cm^2^ zinc chromate (Figure 3A).

We found that XRCC2 foci formation increased after 24 h of Cr(VI) exposure, as 0, 0.1, 0.2, and 0.3 μg/cm^2^ zinc chromate produced 466, 537, 630, and 676 total foci, respectively (Figure 3B). Conversely, after 120 h of zinc chromate exposure, total foci were fewer than those of the control group (Figure 3B). For example, 120 h of exposure to 0, 0.1, 0.2, and 0.3 μg/cm^2^ zinc chromate induced 470, 345, 187, and 108 total foci in 100 cells, respectively.

The remaining member of the BCDX2 complex, RAD51D, was affected differently from the others. Specifically, particulate Cr(VI) inhibited RAD51D foci formation after both 24 and 120 h exposures (Figure 3C). We found that 24 h of exposure to 0, 0.1, 0.2, and 0.3 μg/cm^2^ zinc chromate induced 583, 625, 387, and 236 total RAD51D foci, respectively (Figure 3C). Similarly, 120 h exposure to 0, 0.1, 0.2, and 0.3 μg/cm^2^ zinc chromate induced 744, 626, 332, and 232 total RAD51D foci, respectively (Figure 3C).

### 3.4. Acute and Prolonged Cr(VI) Exposures Decrease RAD51D Protein Levels

**Figure 4.**
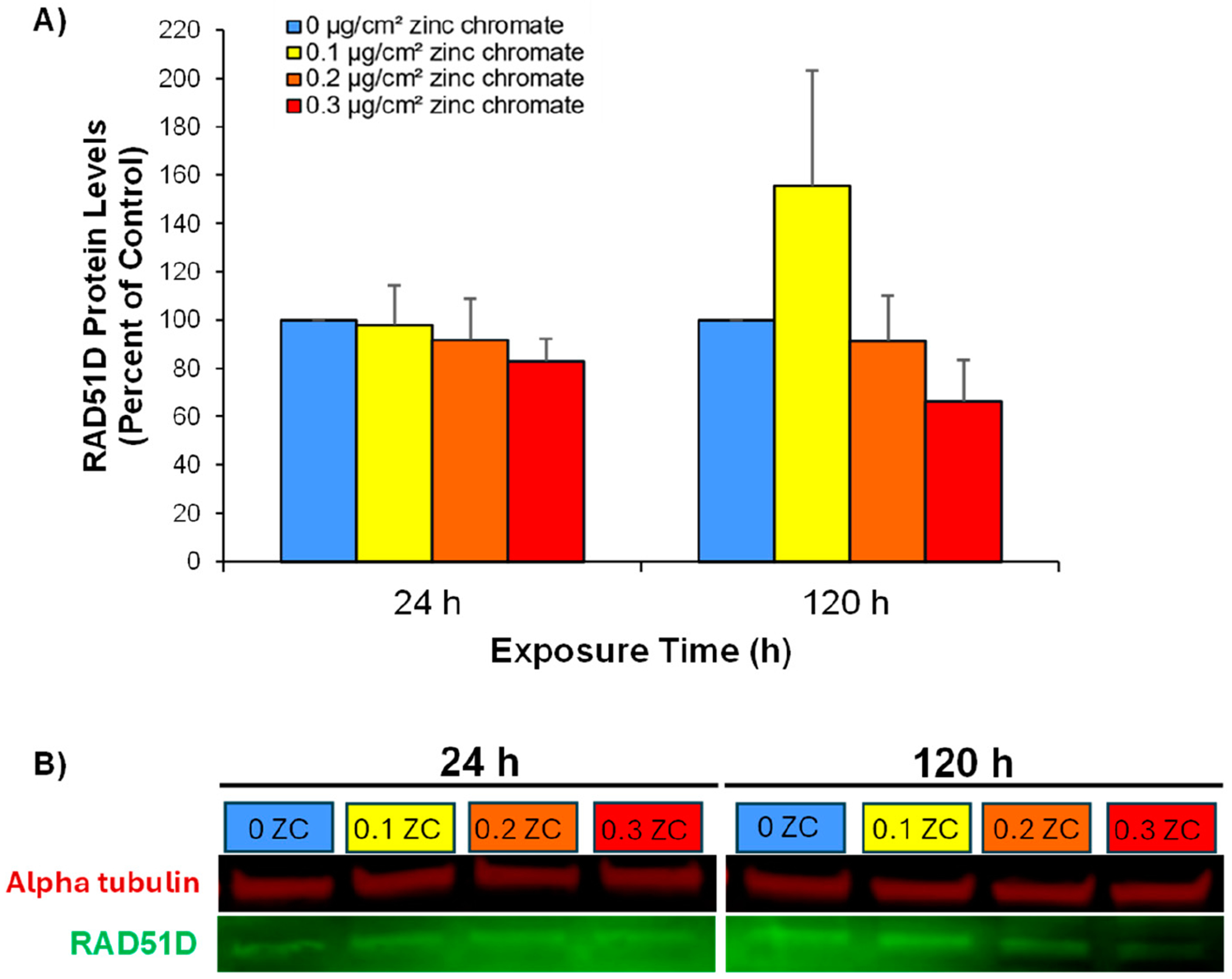
Acute and prolonged exposures to particulate Cr(VI) inhibit RAD51D protein expression. This figure shows that both acute and prolonged zinc chromate exposures reduced RAD51D protein levels in a time- and concentration-dependent manner. (**A**) The graph shows RAD51D protein levels as percent of the control. Data represent the average from three independent experiments ± standard error of the mean. (**B**) Representative western blot image of RAD51D (green) and the loading control alpha-tubulin (red). These images were cropped for the publication; the original images are provided in the Supplementary Materials.

Since RAD51D is the first paralog whose foci are inhibited after early exposures of 24 h, its inhibition may represent the initial target of the Cr(VI)-induced inhibition of homologous recombination repair. Thus, we further explored whether the absence of RAD51D foci was a result of reduced protein expression; whole-cell RAD51D protein levels were measured via western blotting. Particulate Cr(VI) led to a concentration-dependent decrease in RAD51D protein levels at both 24 and 120 h (Figure 4A,B). After 24 h, RAD51D protein levels decreased to 97.8%, 91.6%, and 82.3% of the control at 0.1, 0.2, and 0.3 μg/cm^2^ zinc chromate, respectively. A more pronounced reduction was observed after 120 h, with 0.2, and 0.3 μg/cm^2^ zinc chromate lowering protein levels to 91.2% and 66.2%, respectively.

### 3.5. Particulate Cr(VI) Exposure Fails to Increase Interactions Between RAD51 and RAD51D

**Figure 5.**
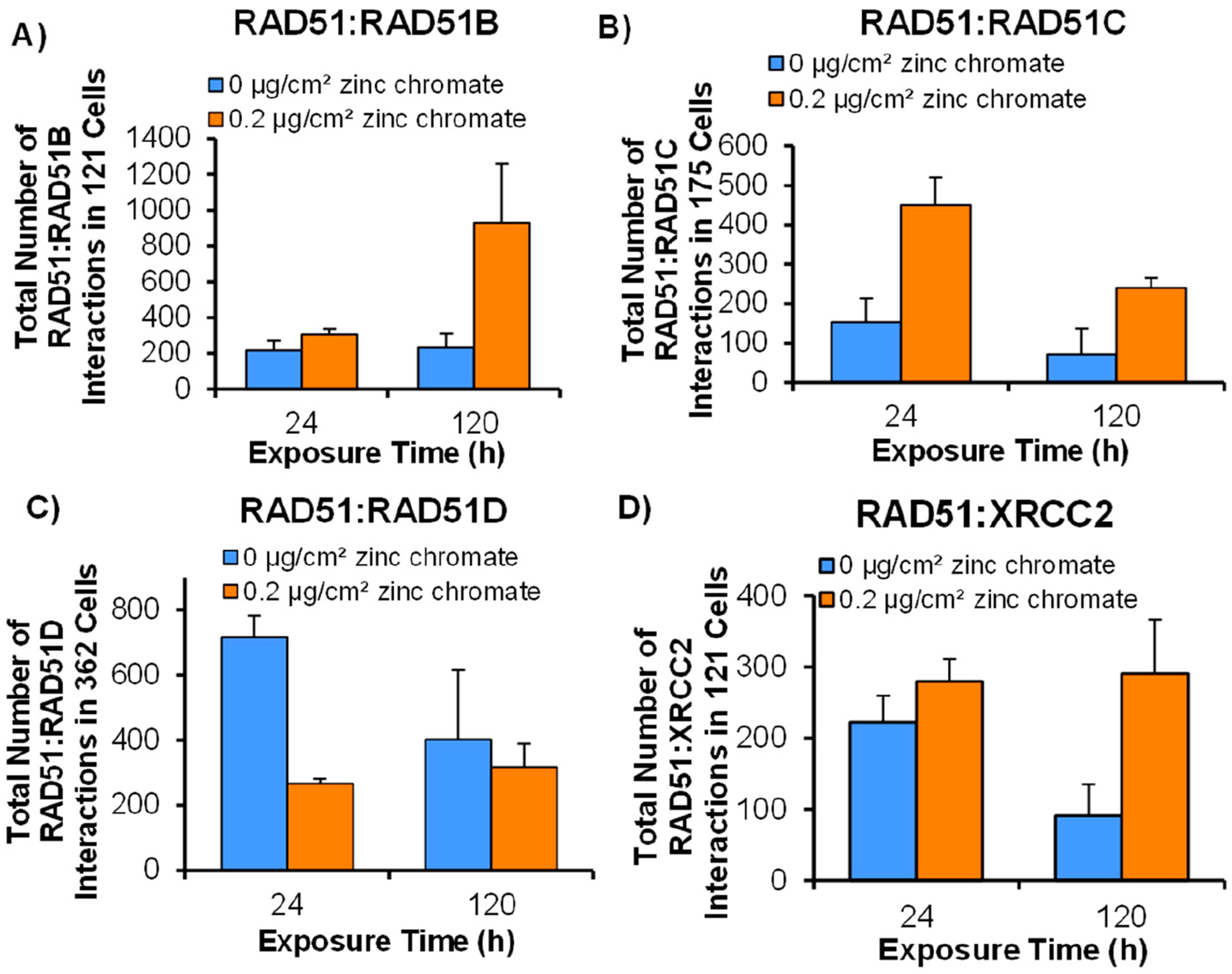
Particulate Cr(VI) exposure induces protein interactions between RAD51 and RAD51B, RAD51C, and XRCC2 but inhibits the interaction with RAD51D. This figure shows the numbers of RAD51 protein–protein interactions with the BCDX2 complex using the Doulink^™^ Proximity Ligation Assay after 24 h and 120 h exposures to 0.2 μg/cm^2^ particulate Cr(VI). Data represent the average from three independent experiments ± standard error of the mean. Brackets show timepoint comparisons. (**A**) RAD51:RAD51B protein interactions; (**B**) RAD51:RAD51C protein interactions; (**C**) RAD51:RAD51D protein interactions; (**D**) RAD51:XRCC2 protein interactions.

Because Cr(VI) increases DNA double strand breaks and homologous recombination is the principal repair pathway, one would expect interactions between RAD51 and the paralogs in the BCDX2 complex to increase as more repair is needed to manage the breaks. However, Cr(VI) targets RAD51 and reduces the amount and complexity of RAD51 nucleoprotein filament formation, suggesting that Cr(VI) first targets the paralogs in the BCDX2 complex, resulting in these effects of RAD51 on the filament. Above, we considered Cr(VI) effects on foci levels for each paralog and found that RAD51D is the first target. Here, we determine the effects of acute (24 h) and prolonged (120 h) Cr(VI) exposures on protein–protein interactions between RAD51 and each of the paralogs. To this end, we measured the co-localization of RAD51 with each paralog using the Duolink^™^ PLA. We found that RAD51/RAD51D foci were inhibited, particularly after 24 h of zinc chromate exposure, while after 120 h of exposure, the trends seemed to be inhibited but did not achieve statistical significance. The interaction did not increase consistently with the loss of RAD51D foci. By contrast, for RAD51 interactions with RAD51B, RAD51C, and RAD51D, we found increases in interaction levels (Figure 5A–D).

### 3.6. Acute and Prolonged Exposures to Cr(VI) Induce RPA Foci

**Figure 6.**
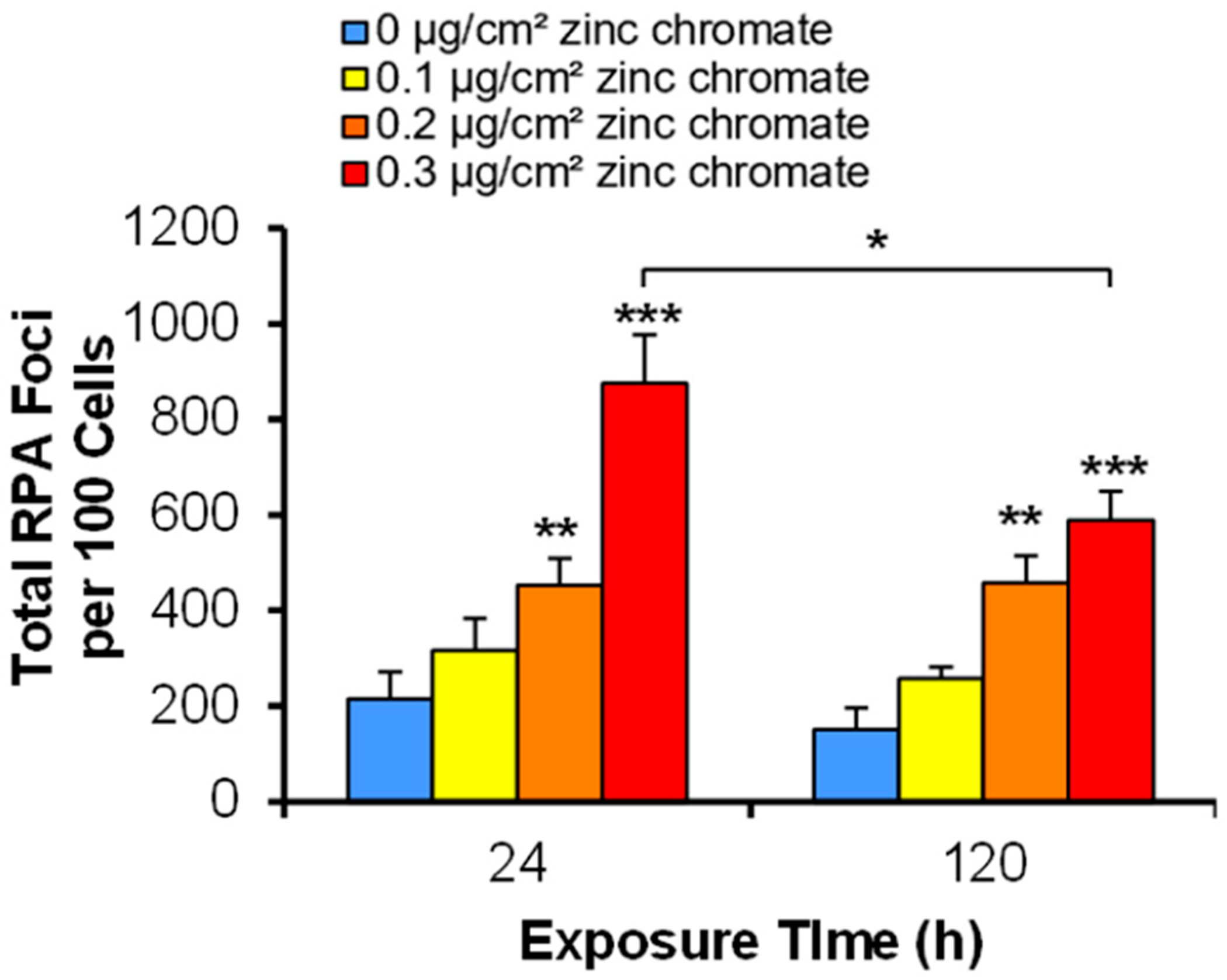
Acute and prolonged Cr(VI) exposures induce RPA foci formation. This figure shows that acute and prolonged zinc chromate exposures reduced RPA foci formation. Results shown are based on total foci. Data represent the average from three independent experiments ± standard error of the mean. * Statistically significant compared to control (*p* < 0.1); ** statistically significant compared to control (*p* < 0.05); *** statistically significant compared to control (*p* < 0.01); brackets show timepoint comparisons.

Replication Protein A (RPA) is essential for homologous recombination repair, as it coats the resected single-stranded DNA to avoid its degradation. This step occurs before the actions of the BCDX2 and CX3 complexes, and thus, we examined whether the pathway is active until this point, which would indicate that the complexes and not the initial steps of homologous recombination repair are targeted by Cr(VI). We found that RPA foci formation increased after 24 h of zinc chromate exposure as 0, 0.1, 0.2, and 0.3 μg/cm^2^ zinc chromate produced 215, 316, 452, and 875 total foci, respectively (Figure 6). After 120 h of zinc chromate exposure, total foci were also significantly increased compared to the control, as 0, 0.1, 0.2, and 0.3 μg/cm^2^ zinc chromate produced 151, 258, 458, and 589 total foci, respectively (Figure 6).

## 4. Discussion

Despite its known toxicity, the exact mechanism by which Cr(VI) leads to cancer remains to be elucidated. Notably, Cr(VI) reduces RAD51 nucleoprotein filament formation, which begins with the loading of RPA, followed by the loading of the BCDX2 complex, which in turn loads RAD51 onto the filament, which is then stabilized by the CX3 complex. This report is the first to assess the impact of Cr(VI) on RPA, the members of the BCDX2 complex, and the members of the CX3 complex. Our results are consistent with studies in Chinese hamster cells deficient in either XRCC3 or RAD51C cells that showed that Cr(VI) increases cytotoxicity, chromosomal aberrations, and complex chromosome aberrations, relative to wild-type cells [53,54], establishing the BCDX2 complex as an important player in the repair of Cr(VI)-induced DNA breaks. We found that RPA foci formation increased as expected in response to both acute and prolonged Cr(VI) exposures in human cells. Contrary to that normal response, our data showed that Cr(VI) exposure led to the suppression of RAD51D. We found that both acute and prolonged Cr(VI) exposures inhibited the RAD51D repair response, as indicated by reduced RAD51D foci formation, lower protein levels, decreased gene expression, and a lack of increased protein interaction with RAD51. We also found that prolonged exposure inhibited foci formation of RAD51B, RAD51C, and XRCC2. These data indicate that Cr(VI) targets the BCDX2 complex, which would affect its loading onto the filament. Cr(VI) exposure also had an effect on the CX3 complex, as acute exposure failed to increase RAD51C or XRCC3 foci, suggesting that there is a loss of stabilization as well.

Our findings suggest that RAD51D is the initial target of Cr(VI), followed by RAD51B, RAD51C, and XRCC2. Our results show that Cr(VI) inhibits RAD51D mRNA, protein, and foci, both after acute and prolonged exposures, and inhibits protein interactions with RAD51. Indeed, our PLA results showed that RAD51 interactions with paralogs RAD51B, RAD51C, and XRCC2 were induced, while interactions between RAD51 and RAD51D were inhibited. This loss of RAD51–RAD51D interactions could be indicative of a loss of RAD51D protein and foci around DNA repair sites. The loss of RAD51D is particularly problematic in homologous recombination repair and promoting genomic stability [62–64]. Sigurdsson et al. showed that the BCDX2 complex forms two subcomplexes: BC and DX2 [65]. Data indicate that the DX2 subcomplex plays a more crucial role than the BC subcomplex in stabilizing the RAD51 filament [47], which aligns with our findings demonstrating that RAD51D is the primary target of Cr(VI) exposure. The loss of RAD51B, RAD51C, RAD51D, and XRCC2 with prolonged exposure is expected to exacerbate the RAD51 loss on the filament. Such a conclusion is consistent with Chun et al., showing that the double depletion of components of the BCDX2 complex leads to a significant reduction of RAD51 foci formation [47]. On the contrary, RAD51D rescuing leads to RAD51 foci formation, indicating the tight relationship between these two repair proteins [66]. In addition, the loss of RAD51D alone is known to promote a shift in DNA repair mechanisms towards single-strand annealing and end joining processes that lead to large chromosomal deletions surrounding DNA double strand break sites, which is consistent with Cr(VI) inducing chromosome instability [67,68].

The mechanism by which Cr(VI) impairs RAD51D is unclear. Our findings showed that RAD51D protein expression decreased after both acute and prolonged exposures. This reduction in RAD51D protein levels may be a result of either decreased protein synthesis or increased protein degradation. However, the observed decrease in RAD51D mRNA expression following both exposures suggests that the most likely explanation is a loss of protein synthesis. Alternatively, Cr ions may bind to associated regulatory proteins, preventing transcription factors from activating. This binding would result in decreased transcriptional activity, lowering the synthesis of RAD51 and the BCDX2 complex. As a result, a reduction in the overall availability of these proteins would impair the cell’s ability to efficiently repair DNA through homologous recombination, leading to increased sensitivity to DNA damage [47,69]. Cr binding may also be disrupting the interaction between RAD51 and RAD51D by inducing structural or conformational changes in RAD51 or RAD51D, preventing their proper binding and coordination. Without this interaction, RAD51’s ability to form nucleoprotein filaments and initiate the homologous strand exchange would be compromised, leading to deficient DNA repair and increased genomic instability [70].

Overall, our findings refine the mechanism of Cr(VI) carcinogenesis by showing that Cr(VI) initially targets RAD51D, which disrupts the BCDX2 complex and its interactions with RAD51 levels. This reduction impairs the homologous recombination repair response, leading to chromosome instability and, ultimately, the development of cancer.

## 5. Conclusions

In summary, this study shows that particulate hexavalent chromium initially targets RAD51D within the BCDX2 complex, a critical component of the homologous recombination repair pathway, followed by impacts on each member of the complex. The interaction between Cr(VI) and RAD51D disrupts the BCDX2 complex’s ability to accurately repair the DNA. Our findings offer valuable insights into the molecular mechanisms underlying Cr(VI)-induced carcinogenesis. Further work will investigate the molecular mechanism for how Cr(VI) disrupts RAD51D.

## Supplementary Material

Supplementary

The following supporting information can be downloaded at: https://www.mdpi.com/article/10.3390/occuphealth1010013/s1. Figure S1: Alpha tubulin loading control.

## Data Availability

The data that support the findings of this study are available from the corresponding author, upon reasonable request.

## Abbreviations

The following abbreviations are used in this manuscript:

BCDX2: RAD51B–RAD51C–RAD51D–XRCC2 complex
CHO: Chinese Hamster Ovary cells
Cr: Chromium
Cr(VI): Hexavalent chromium
CX3: RAD51C-XRCC3 complex
DAPI: 4’6,-diamidino-2-phenylindole
DMEM/F-12: Dulbecco’s Modified Eagle Medium/Nutrient Mixture F-12
DNA: Deoxyribonucleic acid
hTERT: Human telomerase reverse transcriptase
mRNA: Messenger RNA
PLA: Proximity Ligation Assay
RAD51: RAD51 recombinase (key homologous recombination DNA repair protein)
RAD51B: RAD51 Paralog B (a member of the RAD51 paralog family)
RAD51C: RAD51 Paralog C (a member of the RAD51 paralog family)
RAD51D: RAD51 Paralog D (a member of the RAD51 paralog family)
RNA: Ribonucleic acid
RNA-Seq: RNA Sequencing
RPA: Replication Protein A
RT-qPCR: Real-Time Quantitative Polymerase Chain Reaction
WTHBF-6: h-TERT Immortalized Human Bronchial Fibroblasts
XRCC2: X-ray Repair Cross-Complementing 2 (a member of the RAD51 paralog family)
XRCC3: X-ray Repair Cross-Complementing 3 (a member of the RAD51 paralog family)

## References

[1] Spatola C; Militello C; Tocco A; Salamone V; Raffaele L; Migliore M; Pagana A; Milazzotto R; Chillura I; Pergolizzi S; Intensity-modulated radiotherapy for relapsed malignant pleural mesothelioma. Future Oncol. 2016, 12, 67–71. 27651129 10.2217/fon-2016-0330

[2] Chakraborty S; Rahman T The difficulties in cancer treatment. Ecancermedicalscience 2012, 6, ed16. 24883085 10.3332/ecancer.2012.ed16 PMC4024849

[3] Chen QY; DesMarais T; Costa M Metals and Mechanisms of Carcinogenesis. Annu. Rev. Pharmacol. Toxicol 2019, 59, 537–554. 30625284 10.1146/annurev-pharmtox-010818-021031 PMC6348465

[4] Willers H; Azzoli CG; Santivasi WL; Xia F Basic mechanisms of therapeutic resistance to radiation and chemotherapy in lung cancer. Cancer J. 2013, 19, 200–207. 23708066 10.1097/PPO.0b013e318292e4e3 PMC3668666

[5] Imaida K; Yokohira M; Hashimoto N; Kuno T Risk analysis of environmental chemicals on lung carcinogenesis. Asian Pac. J. Cancer Prev 2010, 11, 9–12. 20593918

[6] Heng WS; Kruyt FAE; Cheah SC Understanding Lung Carcinogenesis from a Morphostatic Perspective: Prevention and Therapeutic Potential of Phytochemicals for Targeting Cancer Stem Cells. Int. J. Mol. Sci 2021, 22, 5697. 34071790 10.3390/ijms22115697 PMC8198077

[7] Walter M; Schenkeveld WDC; Tomatis M; Schelch K; Peter-Vörösmarty B; Geroldinger G; Gille L; Bruzzoniti MC; Turci F; Kraemer SM; The Potential Contribution of Hexavalent Chromium to the Carcinogenicity of Chrysotile Asbestos. Chem. Res. Toxicol 2022, 35, 2335–2347. 36410050 10.1021/acs.chemrestox.2c00314 PMC9768810

[8] IARC. Working Group on the Evaluation of Carcinogenic Risks to Humans. In Chromium, Nickel and Welding; IARC Monographs on the Evaluation of Carcinogenic Risks to Humans, No. 49; International Agency for Research on Cancer: Lyon, France, 1990. Available online: https://www.ncbi.nlm.nih.gov/books/NBK519250/ (accessed on 12 December 2025).

[9] IARC. Working Group on the Evaluation of Carcinogenic Risks to Humans. In Arsenic, Metals, Fibres and Dusts; IARC Monographs on the Evaluation of Carcinogenic Risks to Humans, No. 100C; International Agency for Research on Cancer: Lyon, France, 2012. Available online: https://www.ncbi.nlm.nih.gov/books/NBK304375/ (accessed on 12 December 2025). PMC4781271 23189751

[10] Driscoll T; Steenland K; Pruss-UStun A; Imel Nelson D; Leigh J Occupational Carcinogens: Assessing the Environmental Burden of Disease at National and Local Levels; Environmental Burden of Disease Series No. 6; World Health Organization: Geneva, Switzerland, 2004.

[11] Mannetje A; Bencko V; Brennan P; Zaridze D; Szeszenia-Dabrowska N; Rudnai P; Lissowska J; Fabiánová E; Cassidy A; Mates D; Occupational exposure to metal compounds and lung cancer. Results from a multi-center case-control study in Central/Eastern Europe and UK. Cancer Causes Control 2011, 22, 1669–1680. 21960145 10.1007/s10552-011-9843-3

[12] Cherrie JW; Hutchings S; Gorman Ng M; Mistry R; Corden C; Lamb J; Sánchez Jiménez A; Shafrir A; Sobey M; van Tongeren M; Prioritising action on occupational carcinogens in Europe: A socioeconomic and health impact assessment. Br. J. Cancer 2017, 117, 274–281. 28609433 10.1038/bjc.2017.161 PMC5520511

[13] Reif BM; Murray BP Chromium Toxicity. In StatPearls; StatPearls Publishing: Treasure Island, FL, USA, 2025. Available online: https://www.ncbi.nlm.nih.gov/books/NBK599502/ (accessed on 11 January 2024).

[14] IARC Monographs on the Evaluation of Carcinogenic Risks to Humans. In Welding, Molybdenum Trioxide, and Indium Tin Oxide; International Agency for Research on Cancer: Lyon, France, 2018. 31268644

[15] Kieber RJ; Willey JD; Zvalaren SD Chromium speciation in rainwater: Temporal variability and atmospheric deposition. Environ. Sci. Technol 2002, 36, 5321–5327. 12521156 10.1021/es020777n

[16] Pappas RS Toxic elements in tobacco and in cigarette smoke: Inflammation and sensitization. Metallomics 2011, 3, 1181–1198. 21799956 10.1039/c1mt00066g PMC4542087

[17] Yu CH; Huang L; Shin JY; Artigas F; Fan ZH Characterization of concentration, particle size distribution, and contributing factors to ambient hexavalent chromium in an area with multiple emission sources. Atmos. Environ 2014, 94, 701–708. 10.1016/j.atmosenv.2014.06.004 PMC4351811 25750579

[18] Hess CA; Olmedo P; Navas-Acien A; Goessler W; Cohen JE; Rule AM E-cigarettes as a source of toxic and potentially carcinogenic metals. Environ. Res 2017, 152, 221–225. 27810679 10.1016/j.envres.2016.09.026 PMC5135636

[19] Sasaki H; Takahashi T; Futami M; Endo T; Hirano M; Kotake Y; Pham K-O Airborne Microplastics: Source Implications from Particulate Matter Composition. Atmosphere 2025, 16, 1222.

[20] Zhigalenok Y; Tazhibayeva A; Kokhmetova S; Starodubtseva A; Kan T; Isbergenova D; Malchik F Hexavalent chromium at the crossroads of science, environment and public health. RSC Adv. 2025, 15, 21439–21464. 40567474 10.1039/d5ra03104d PMC12188526

[21] Ukhurebor KE; Aigbe UO; Onyancha RB; Nwankwo W; Osibote OA; Paumo HK; Ama OM; Adetunji CO; Siloko IU Effect of hexavalent chromium on the environment and removal techniques: A review. J. Environ. Manag 2021, 280, 111809. 10.1016/j.jenvman.2020.111809 33360556

[22] Hirose T; Kondo K; Takahashi Y; Ishikura H; Fujino H; Tsuyuguchi M; Hashimoto M; Yokose T; Mukai K; Kodama T; Frequent microsatellite instability in lung cancer from chromate-exposed workers. Mol. Carcinog 2002, 33, 172–180. 11870883 10.1002/mc.10035

[23] Wise SS; Aboueissa AE; Martino J; Wise JP Sr. Hexavalent Chromium-Induced Chromosome Instability Drives Permanent and Heritable Numerical and Structural Changes and a DNA Repair-Deficient Phenotype. Cancer Res. 2018, 78, 4203–4214. 29880483 10.1158/0008-5472.CAN-18-0531 PMC6072558

[24] Proctor DM; Suh M; Campleman SL; Thompson CM Assessment of the mode of action for hexavalent chromium-induced lung cancer following inhalation exposures. Toxicology 2014, 325, 160–179. 25174529 10.1016/j.tox.2014.08.009

[25] DesMarais TL; Costa M Mechanisms of Chromium-Induced Toxicity. Curr. Opin. Toxicol 2019, 14, 1–7. 31511838 10.1016/j.cotox.2019.05.003 PMC6737927

[26] Qin Q; Xie H; Wise SS; Browning CL; Thompson KN; Holmes AL; Wise JP Sr. Homologous recombination repair signaling in chemical carcinogenesis: Prolonged particulate hexavalent chromium exposure suppresses the Rad51 response in human lung cells. Toxicol. Sci 2014, 142, 117–125. 25173789 10.1093/toxsci/kfu175 PMC4271064

[27] Browning CL; Wise JP Sr. Prolonged exposure to particulate chromate inhibits RAD51 nuclear import mediator proteins. Toxicol. Appl. Pharmacol 2017, 331, 101–107. 28554658 10.1016/j.taap.2017.05.030 PMC5568470

[28] Meaza I; Williams AR; Lu H; Kouokam JC; Toyoda JH; Croom-Perez TJ; Wise SS; Aboueissa AE; Wise JP Sr. Prolonged particulate hexavalent chromium exposure induces RAD51 foci inhibition and cytoplasmic accumulation in immortalized and primary human lung bronchial epithelial cells. Toxicol. Appl. Pharmacol 2023, 479, 116711. 37805091 10.1016/j.taap.2023.116711 PMC10841504

[29] Lu H; Toyoda JH; Wise SS; Browning CL; Speer RM; Croom-Pérez TJ; Bolt A; Meaza I; Wise JP Sr. A whale of a tale: Whale cells evade the driving mechanism for hexavalent chromium-induced chromosome instability. Toxicol. Sci 2024, 199, 49–62. 38539048 10.1093/toxsci/kfae030 PMC11057468

[30] Xie H; Holmes AL; Young JL; Qin Q; Joyce K; Pelsue SC; Peng C; Wise SS; Jeevarajan AS; Wallace WT; Zinc chromate induces chromosome instability and DNA double strand breaks in human lung cells. Toxicol. Appl. Pharmacol 2009, 234, 293–299. 19027772 10.1016/j.taap.2008.10.010 PMC4075174

[31] Brooks B; O’Brien TJ; Ceryak S; Wise JP Sr.; Wise SS; Wise JP Jr.; Defabo E; Patierno SR Excision repair is required for genotoxin-induced mutagenesis in mammalian cells. Carcinogenesis 2008, 29, 1064–1069. 18332048 10.1093/carcin/bgn058 PMC2902384

[32] Browning CL; Qin Q; Kelly DF; Prakash R; Vanoli F; Jasin M; Wise JP Sr. Prolonged Particulate Hexavalent Chromium Exposure Suppresses Homologous Recombination Repair in Human Lung Cells. Toxicol. Sci 2016, 153, 70–78. 27449664 10.1093/toxsci/kfw103 PMC5601504

[33] Meaza I; Williams AR; Wise SS; Lu H; Wise JP Sr. Carcinogenic Mechanisms of Hexavalent Chromium: From DNA Breaks to Chromosome Instability and Neoplastic Transformation. Curr. Environ. Health Rep 2024, 11, 484–546. 39466546 10.1007/s40572-024-00460-9 PMC11872169

[34] Lu H; Wise SS; Toyoda JH; Speer RM; Croom-Perez TJ; Meaza I; Kouokam JC; Wise JL; Hoyle G; Chen N; Particulate hexavalent chromium exposure induces DNA double-strand breaks and inhibits homologous recombination repair in rat and human lung tissues. Chemosphere 2025, 370, 143982. 39701314 10.1016/j.chemosphere.2024.143982 PMC11750071

[35] Wise JTF; Lu H; Meaza I; Wise SS; Williams AR; Wise JY; Mason MD; Wise JP Sr. Prolonged Particulate Hexavalent Chromium Exposure Induces DNA Double-Strand Breaks and Inhibits Homologous Recombination Repair in Primary Rodent Lung Cells. Biol. Trace Elem. Res 2024, 202, 5653–5663. 38499919 10.1007/s12011-024-04136-1 PMC11408706

[36] Haberland VMM; Magin S; Iliakis G; Hartwig A Impact of Manganese and Chromate on Specific DNA Double-Strand Break Repair Pathways. Int. J. Mol. Sci 2023, 24, 10392. 37373538 10.3390/ijms241210392 PMC10298927

[37] Andriuskevicius T; Kotenko O; Makovets S Putting together and taking apart: Assembly and disassembly of the Rad51 nucleoprotein filament in DNA repair and genome stability. Cell Stress 2018, 2, 96–112. 31225474 10.15698/cst2018.05.134 PMC6551702

[38] Meyer D; Gore SK; Liu J; Ceballos SJ; Hung S-H; Reginato G; Cano-Linares MI; Maslowska KH; Villafañez F; Fasching C; Rad51 determines pathway usage in post-replication repair. Nat. Commun 2026, 17, 1359. 41519855 10.1038/s41467-025-68109-1 PMC12876063

[39] Foertsch F; Kache T; Drube S; Biskup C; Nasheuer HP; Melle C Determination of the number of RAD51 molecules in different human cell lines. Cell Cycle 2019, 18, 3581–3588. 31731884 10.1080/15384101.2019.1691802 PMC6927726

[40] Deveryshetty J; Mistry A; Pangeni S; Ghoneim M; Tokmina-Lukaszewska M; Gore SK; Liu J; Kaushik V; Karunakaran S; Taddei A; Mechanism of Rad51 filament formation by Rad52 and Rad55-Rad57 in homologous recombination. Nat. Commun 2025, 16, 6685. 40691144 10.1038/s41467-025-61811-0 PMC12280074

[41] Sullivan MR; Bernstein KA RAD-ical New Insights into RAD51 Regulation. Genes 2018, 9, 629. 30551670 10.3390/genes9120629 PMC6316741

[42] Grundy MK; Buckanovich RJ; Bernstein KA Regulation and pharmacological targeting of RAD51 in cancer. NAR Cancer 2020, 2, zcaa024. 33015624 10.1093/narcan/zcaa024 PMC7520849

[43] Bonilla B; Hengel SR; Grundy MK; Bernstein KA RAD51 Gene Family Structure and Function. Annu. Rev. Genet 2020, 54, 25–46. 32663049 10.1146/annurev-genet-021920-092410 PMC7703940

[44] Schild D; Lio YC; Collins DW; Tsomondo T; Chen DJ Evidence for simultaneous protein interactions between human Rad51 paralogs. J. Biol. Chem 2000, 275, 16443–16449. 10749867 10.1074/jbc.M001473200

[45] Masson JY; Tarsounas MC; Stasiak AZ; Stasiak A; Shah R; McIlwraith MJ; Benson FE; West SC Identification and purification of two distinct complexes containing the five RAD51 paralogs. Genes Dev. 2001, 15, 3296–3307. 11751635 10.1101/gad.947001 PMC312846

[46] Yonetani Y; Hochegger H; Sonoda E; Shinya S; Yoshikawa H; Takeda S; Yamazoe M Differential and collaborative actions of Rad51 paralog proteins in cellular response to DNA damage. Nucleic Acids Res. 2005, 33, 4544–4552. 16093548 10.1093/nar/gki766 PMC1184222

[47] Chun J; Buechelmaier ES; Powell SN Rad51 paralog complexes BCDX2 and CX3 act at different stages in the BRCA1-BRCA2-dependent homologous recombination pathway. Mol. Cell. Biol 2013, 33, 387–395. 23149936 10.1128/MCB.00465-12 PMC3554112

[48] Greenhough LA; Liang CC; Belan O; Kunzelmann S; Maslen S; Rodrigo-Brenni MC; Anand R; Skehel M; Boulton SJ; West SC Structure and function of the RAD51B-RAD51C-RAD51D-XRCC2 tumour suppressor. Nature 2023, 619, 650–657. 37344587 10.1038/s41586-023-06179-1 PMC7614784

[49] Rawal Y; Jia L; Meir A; Zhou S; Kaur H; Ruben EA; Kwon Y; Bernstein KA; Jasin M; Taylor AB; Structural insights into BCDX2 complex function in homologous recombination. Nature 2023, 619, 640–649. 37344589 10.1038/s41586-023-06219-w PMC10712684

[50] Koo CW; Xiao J; Coassolo S; Liu J; Yu C; Azumaya C; Gore SK; Cheung TK; Brillantes B; Rose CM; BCDX2-CX3 and DX2-CX3 complexes assemble and stabilize RAD51 filaments. Nature 2026, ahead of print. 10.1038/s41586-026-10314-z PMC13537095 41772053

[51] Thrasher JG; Fagunloye AAG; Justiniano FS; Bernstein KA; Jensen RB RAD51 Paralogs and RAD51 Paralog Complexes BCDX2 and CX3 Interact with BRCA2. bioRxiv 2024.

[52] Sahoo S; Nagraj T; Bhattacharya D; Nagar N; Somyajit K; Poluri KM; Nagaraju G RAD51C-XRCC3 complex regulates FANCM-mediated R-loop resolution to safeguard genome integrity. Sci. Adv 2026, 12, eaea5932. 41719405 10.1126/sciadv.aea5932 PMC12922753

[53] Bryant HE; Ying S; Helleday T Homologous recombination is involved in repair of chromium-induced DNA damage in mammalian cells. Mutat. Res 2006, 599, 116–123. 16564059 10.1016/j.mrfmmm.2006.02.001

[54] Stackpole MM; Wise SS; Goodale BC; Duzevik EG; Munroe RC; Thompson WD; Thacker J; Thompson LH; Hinz JM; Wise JP Sr. Homologous recombination repair protects against particulate chromate-induced chromosome instability in Chinese hamster cells. Mutat. Res 2007, 625, 145–154. 17662313 10.1016/j.mrfmmm.2007.06.003 PMC2230547

[55] Wise SS; Elmore LW; Holt SE; Little JE; Antonucci PG; Bryant BH; Wise JP Sr. Telomerase-mediated lifespan extension of human bronchial cells does not affect hexavalent chromium-induced cytotoxicity or genotoxicity. Mol. Cell. Biochem 2004, 255, 103–111. 14971651 10.1023/b:mcbi.0000007266.82705.d9

[56] Martino J; Holmes AL; Xie H; Wise SS; Wise JP Sr. Chronic Exposure to Particulate Chromate Induces Premature Centrosome Separation and Centriole Disengagement in Human Lung Cells. Toxicol. Sci 2015, 147, 490–499. 26293554 10.1093/toxsci/kfv146 PMC4635651

[57] Meaza I; Cahill CR; Speer RM; Kouokam JC; Wise JP Sr. Particulate hexavalent chromium inhibits global transcription of genes in DNA repair pathways, particularly targeting homologous recombination repair, base excision repair, mismatch repair and microhomology-mediated end-joining. J. Hazard. Mater 2025, 485, 136892. 39706010 10.1016/j.jhazmat.2024.136892 PMC11794018

[58] Speer RM; Meaza I; Toyoda JH; Lu Y; Xu Q; Walter RB; Kong M; Lu H; Kouokam JC; Wise JP Sr. Particulate hexavalent chromium alters microRNAs in human lung cells that target key carcinogenic pathways. Toxicol. Appl. Pharmacol 2022, 438, 115890. 35101437 10.1016/j.taap.2022.115890 PMC8938933

[59] Xie H; Wise SS; Holmes AL; Xu B; Wakeman TP; Pelsue SC; Singh NP; Wise JP Sr. Carcinogenic lead chromate induces DNA double-strand breaks in human lung cells. Mutat. Res 2005, 586, 160–172. 16112599 10.1016/j.mrgentox.2005.06.002 PMC4136752

[60] Toyoda JH; Martino J; Speer RM; Meaza I; Lu H; Williams AR; Bolt AM; Kouokam JC; Aboueissa AE; Wise JP Sr. Hexavalent Chromium Targets Securin to Drive Numerical Chromosome Instability in Human Lung Cells. Int. J. Mol. Sci 2023, 25, 256. 38203427 10.3390/ijms25010256 PMC10778806

[61] Meaza I; Wise JL; Wise SS; Lu H; Williams AR; Delnicki M; Easley J; Kouokam JC; Wise JP Jr.; Vielee ST; Oropharyngeal aspiration of particulate hexavalent chromium increases chromium levels in lung and liver, and induces essential metal dyshomeostasis in lung, liver, and blood. J. Trace Elem. Med. Biol 2025, 91, 127705. 40773862 10.1016/j.jtemb.2025.127705 PMC12435473

[62] Rajesh P; Litvinchuk AV; Pittman DL; Wyatt MD The homologous recombination protein RAD51D mediates the processing of 6-thioguanine lesions downstream of mismatch repair. Mol. Cancer Res 2011, 9, 206–214. 21205838 10.1158/1541-7786.MCR-10-0451 PMC3041871

[63] Rajesh C; Baker DK; Pierce AJ; Pittman DL The splicing-factor related protein SFPQ/PSF interacts with RAD51D and is necessary for homology-directed repair and sister chromatid cohesion. Nucleic Acids Res. 2011, 39, 132–145. 20813759 10.1093/nar/gkq738 PMC3017596

[64] Slijepcevic P The role of DNA damage response proteins at telomeres—An “integrative” model. DNA Repair 2006, 5, 1299–1306. 16798109 10.1016/j.dnarep.2006.05.038

[65] Sigurdsson S; Van Komen S; Bussen W; Schild D; Albala JS; Sung P Mediator function of the human Rad51B-Rad51C complex in Rad51/RPA-catalyzed DNA strand exchange. Genes Dev. 2001, 15, 3308–3318. 11751636 10.1101/gad.935501 PMC312844

[66] Zafar F; Seidler SB; Kronenberg A; Schild D; Wiese C Homologous recombination contributes to the repair of DNA double-strand breaks induced by high-energy iron ions. Radiat. Res 2010, 173, 27–39. 20041757 10.1667/RR1910.1

[67] Ren L; Yao R; Hou T; Liu C; Zhao F; Chen X; Zhang Z; Huang Y Pan-cancer analysis of homologous recombination deficiency and homologous recombination repair-associated gene alterations in solid tumors from a large Asian cohort. BMC Cancer 2025, 25, 946. 40420266 10.1186/s12885-025-14267-w PMC12107993

[68] Reh WA; Nairn RS; Lowery MP; Vasquez KM The homologous recombination protein RAD51D protects the genome from large deletions. Nucleic Acids Res. 2017, 45, 1835–1847. 27924006 10.1093/nar/gkw1204 PMC5389663

[69] Jin ZL; Kim NH RAD51 maintains chromosome integrity and mitochondrial distribution during porcine oocyte maturation in vitro. J. Reprod. Dev 2017, 63, 489–496. 28757527 10.1262/jrd.2017-078 PMC5649098

[70] Smiraldo PG; Gruver AM; Osborn JC; Pittman DL Extensive chromosomal instability in Rad51d-deficient mouse cells. Cancer Res. 2005, 65, 2089–2096. 15781618 10.1158/0008-5472.CAN-04-2079

