# Supplementary material for: Particulate Hexavalent Chromium Inhibits RAD51 Paralogs Necessary for RAD51 Filament Formation and Stabilization During Homologous Recombination Repair
Source: Occup Health (Auckl). Author manuscript; Available in PMC 2026 May 21. (PMC13189539; doi:10.3390/occuphealth1010013)
Supplement: Supplementary [file NIHMS2170874-supplement-Supplementary.pdf]

# Supplementary material

## Chemicals and Reagents:

Dulbecco's Modified Eagle Medium/Hams F-12 50/50 Mix (DMEM/-12), glutagro, 1X Dulbecco's phosphate-buffered saline (DPBS) without calcium or magnesium, penicillin/streptomycin, sodium pyruvate, and cell cultureware were purchased from Corning, Inc. (Corning, New York). Cosmic calf serum (CCS) was purchased from Hyclone Laboratories, Inc. (Logan, Utah). Trypsin-EDTA (0.25%) was purchased from Gibco (Carlsbad, California). Lab-Tek™ II glass chamber 4 and 8 well slides, 4% paraformaldehyde in PBS, DAPI Diamond, nitrocellulose membranes, HALT protease and phosphatase inhibitor cocktail, RIPA buffer, extraction reagents, a TaqMan Fast Advanced Master Mix Cat#4444557, High-Capacity cDNA Reverse Transcription, TaqMan Assays, and Super Up Rite slides were purchased from Thermo Fisher Scientific (Waltham, MA). FNC coating mix was purchased from AthenaES (Arbutus, Maryland). Sodium azide, Tween-20 and methanol were purchased from VWR (Radnor, Pennsylvania). Duolink® Proximity Ligation Assay (PLA) Multicolor Kits and Triton-X 100 were purchased from Millipore-Sigma (St. Louis, Missouri). Mini-Protean TGX gels were purchased from Bio-Rad Laboratories (Hercules, California). 4X protein sample loading buffer, Odyssey blocking buffer, IRDye® 800CW mouse (90408-07), and IRDye® 680RD Rabbit (90827-21) near-infrared fluorescent secondary antibodies were purchased from Li-Cor Biosciences (Lincoln, Nebraska). Anti-RPA32/RPA2 mouse (Ab-2175) and RAD51C (Ab-55728) primary antibodies were purchased from Abcam (Cambridge, Massachusetts). Anti-RAD51B (NB100-176 A2) and anti-Rad51D (NB100-178 A12) primary antibodies were purchased from Novus Biologicals (Centennial, Colorado). Mouse anti-RAD51D (398819) primary antibodies were purchased from Santa Cruz Biotechnology (Santa Cruz, California). XRCC2 monoclonal antibody (3D9A1 66652-1-IG) was purchased from Proteintech Group, Inc (Rosemont, Illinois). Secondary antibodies, Alexa Fluor 594 (2179864) and Alexa Fluor 488 goat anti-mouse IgG (2066710) were purchased from Thermo Fischer Scientific. Anti-alpha-tubulin, Rabbit (IIHI0) was purchased from Millipore-Sigma. Zinc chromate (CAS# 13530–65-9, 99.7% purity) was purchased from Pfaltz & Bauer, Inc (Waterbury, CT).

## Uncropped representative images of RAD51D western blots.

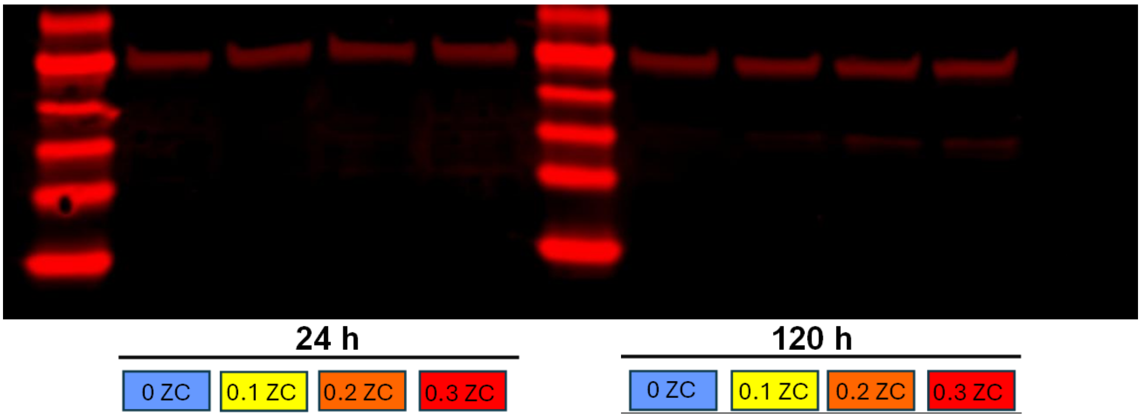


RAD51D target (green):

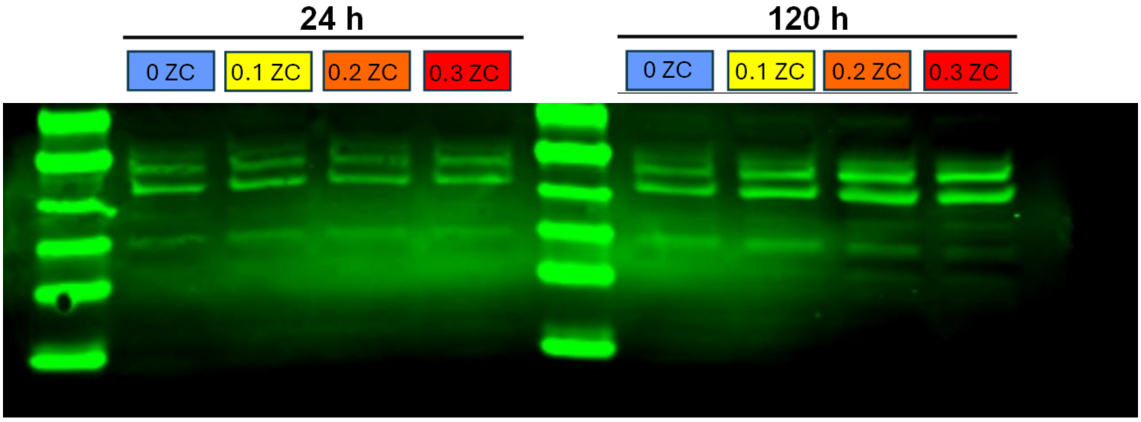


**Figure S1.** Alpha tubulin loading control.
